# Paroxetine-induced hypoglycemia: a clinical case report

**DOI:** 10.1192/j.eurpsy.2025.1314

**Published:** 2025-08-26

**Authors:** A. Decimo, P. Catapano, S. Cipolla, V. Auricchio, G. Capobianco, A. Volpicelli, F. Perris, F. Catapano

**Affiliations:** 1 Psychiatry, University of Campania “Luigi Vanvitelli”, Naples, Italy

## Abstract

**Introduction:**

Major Depressive Disorder (MDD) is a prevalent mental health condition that significantly impairs daily life and increases suicide risk. Selective serotonin reuptake inhibitors (SSRIs), such as paroxetine, are widely prescribed due to their efficacy and favorable safety profile. However, SSRIs can impact glucose metabolism, leading to both hyperglycemia and hypoglycemia, particularly in diabetic patients.

In rare instances, SSRIs have induced hypoglycemia in non-diabetic individuals. Paroxetine, known for its strong inhibition of serotonin reuptake, has been implicated in cases of hypoglycemia, though the precise mechanisms remain unclear.

**Objectives:**

With the present report, we examined wheter the hypotesis that paroxetine induces hypoglycemia is valid.

**Methods:**

This case report discusses an episode of paroxetine-induced hypoglycemia in a 60-year-old female, non-diabetic patient with a history of MDD.

**Results:**

The patient was admitted to our in-patient psychiatric unit due to worsening depressive symptoms and recurrent episodes of sweating, dizziness, tremors, and loss of consciousness, suggestive of hypoglycemic crises. Over the previous three years the patient had been on paroxetine 40 mg/day, thus the drug was suspected as a potential cause of these hypoglycemic episodes and it was discontinued whereas fluoxetine and trazodone were administered. By discharge, her depressive symptoms and insomnia had improved significantly

**Conclusions:**

This case highlights the potential for SSRIs, particularly paroxetine, to induce hypoglycemia even in non-diabetic patients. Although hypoglycemia is typically linked to diabetic treatments, this case demonstrates that antidepressants can also play a role in disrupting glucose regulation. In this case, the symptoms subsided after the discontinuation of paroxetine, strongly suggesting its role in the hypoglycemic episodes, as supported by a &quot;probable&quot; causality score on Naranjo’s scale. Given the nonspecific nature of hypoglycemic symptoms and the risk of misdiagnosis as psychiatric or somatic conditions, it is crucial for healthcare providers to consider medication-related causes. This report underscores the importance of monitoring glycemic levels in patients on SSRIs, particularly when presenting with atypical symptoms. Increased awareness can help prevent misdiagnosis and facilitate timely intervention to avoid severe complications.

**Disclosure of Interest:**

None Declared

